# Integrating substance use peer support and screening brief intervention and referral to treatment services in the emergency department: a descriptive study of the ED leads program

**DOI:** 10.1186/s13722-024-00445-x

**Published:** 2024-02-28

**Authors:** Lynsey Avalone, Monique Lalane, Carla King, Kayna Pfeiffer, Rebecca Linn-Walton, Charles Barron

**Affiliations:** 1 NYC Health + Hospitals/Office of Behavioral Health, 50 Water Street, New York City, NY 10004 USA; 2 NYC Health + Hospitals/Bellevue, 462 1st Avenue, New York City, NY 10016 USA; 3 Mt. Sinai Ichan School of Medicine, 1 Gustave L. Levy Pl, New York City, NY 10029 USA

**Keywords:** Substance use, Emergency medicine, Recovery, Peers, Behavioral health, Addiction

## Abstract

**Background:**

The ED Leads program was introduced to 11 emergency departments (EDs) within New York City public hospitals from 2018 to 2019 to address a need for addiction support services in the ED. The purpose of this study is to (i) describe the ED Leads blended licensed-clinician and peer counselor team model in the ED at three hospitals, (ii) provide a descriptive analysis of patient engagement and referrals to substance use disorder (SUD) care post-intervention, and (iii) highlight potential barriers and facilitators to implementing the model.

**Methods:**

The program intended to combine Screening Brief Intervention and Referral to Treatment and peer support services. The authors analyzed electronic medical records data for patients encountered by ED Leads in the first 120 days of program launch. Data included the outcome of an encounter when a patient was engaged with one or both staff types, and 7-day attendance at an SUD treatment appointment when a patient accepted a referral within the 11-hospital system.

**Results:**

There were 1785 patients approached by ED Leads staff during the study period. Engagement differed by staff type and patient demographics, and encounter outcomes varied significantly by hospital. Eighty-four percent (N = 1503) of patients who were approached engaged with at least one staff type, and 6% (N = 86) engaged with both. Patients were predominantly male (N = 1438, 81%) with an average age of 45 (SD = 13), and enrolled in Medicaid (N = 1062, 59%). A majority (N = 801, 45%) had alcohol use disorder. Of the patients who accepted a referral within the system (N = 433), 63% received treatment services within 7 days of the ED Leads encounter, a majority at detoxification treatment (N = 252, 58%).

**Conclusions:**

This study describes the potential value and challenges of implementing a blended peer counselor and licensed clinician model in the ED to provide SUD services. While teams provided a high volume of referrals and the analysis of post-intervention treatment follow up is promising, the blended team model was not fully realized, making it difficult to assess the benefits of this combined service. Further research might examine patient outcomes among ED patients who are offered services by both a peer counselor and licensed clinician.

## Background

In 2021, 106,699 drug overdose deaths occurred in the United States, an increase of 14% from 2020 [1, 2]. In New York City (NYC), there were 2668 overdose deaths in 2021, increasing 80% since 2019 and 25% since 2020 [3]. Yet, in 2021, the National Survey on Drug Use and Health found only 6.8% of the 43.7 million individuals with an identified need for treatment of a substance use disorder (SUD) had received care within the prior year [4]. Additionally, over the last decade, 87% of people with opioid use disorder (OUD) did not receive medication for opioid use disorder (MOUD) treatment [5].

Individuals with SUD often frequent emergency medical services, which may be their only point of contact with the healthcare system [6]. An emergency department (ED) visit provides a unique and potentially lifesaving opportunity for intervention, and is increasingly recognized as an important setting to identify and engage patients with SUD [6, 7]. However, ED staff have competing priorities, are often overburdened and insufficiently trained to provide interventions for SUDs [8].

To address this service gap, specialized substance use intervention teams have recently been integrated into ED service delivery [8–13]. ED substance use services often incorporate components of screening, brief intervention, and/or referral for ongoing treatment, in alignment with the Substance Abuse and Mental Health Services Administration’s definition of this modality [14]. The literature has had mixed but largely supportive results for screening and subsequent brief intervention in the ED for alcohol use disorders (AUDs), and less promising results for other drug use [6, 14–16]. Across studies, there is a lack of consistency in defining these approaches and in the examination of outcomes (such as a reduction in substance consumption, improved health outcomes, reduction in service utilization, or successful connection to specialized treatment) [15–17]. One study by Krupski et al. did find brief interventions for both alcohol and drug use increased the probability of initiating treatment [18]. Studies by Bohnert et al. and Bonar et al. suggest brief intervention may lead to moderate reductions in substance use and substance related consequences or increasing confidence and intention of seeking help for drug use [19, 20]. Other results find that brief motivation-based interventions may not consistently be effective in the absence of concurrent pharmacotherapy and/or referrals to treatment [21]. Similarly, Bogenshutz et al. found that the brief intervention with a referral did not improve substance use outcomes over the control group receiving an informational packet only [22]. Despite mixed results, the United States Preventative Services Task Force recommends screening for unhealthy drug use when appropriate care and treatment can be provided [23]. To the best of the authors’ knowledge, no study has been conducted to explicity examine or investigate the impact of referral to treatment for those with AUDs [18, 19, 21].

Integration of peer counselors (PCs) in the ED to provide substance use intervention is also promising but understudied [10, 12]. PCs use their lived experience with SUD to support patients and model the process of recovery [8, 24, 25]. PCs provide non-clinical social support (emotional, instrumental, informational, and affiliational) and utilize engagement strategies that differ from more traditional healthcare professionals [13, 26]. PCs in the ED have increased patients’ linkage to care, shortened days to initiation of SUD treatment, and improved engagement of high-risk populations with increased harm reduction education and provision of naloxone [9, 11, 27]. The use of motivational interviewing by PCs in their encounters and when connecting patients to SUD treatment was found to be linked to a 13% decrease in ED visits and 58% decrease in further inpatient medical admissions [28].

Studies describing implementation or effectiveness of an ED intervention that combines licensed masters-level clinician-delivered Screening Brief Intervention and Referral to Treatment (social workers, mental health counselors, and substance use counselors) with PCs are further limited. One study combined PCs with staff who had a bachelor’s degree or higher in the ED and provided patients with an intervention following an opioid overdose. This study, however, did not consider patients with other SUDs in the intervention [8]. Other quantitative studies exclusively focused on patients post opioid-overdose, or utilized a service model with a defined time period for follow-up after intervention by staff who were not employed by the hospital [8, 12, 13]. A qualitative analysis found ED staff were supportive of the concept that teams of licensed clinicians and PCs would provide substance use interventions to ED opioid overdose patients [29]. More evidence supports substance use consult services for hospitalized patients, suggesting research should continue to expand in this area to additional service delivery settings and service models [30–32]. While there has been an impetus to integrate PCs into ED staffing models, there has not been a standardized model or approach identified in previous research [10, 33].

As the rate of drug and alcohol deaths increases, so does political support for substance use interventions at the local, state, and federal level [7, 34]. In response to increasing overdose rates in NYC and the potential effectiveness of ED-SUD interventions, the City of New York provided financial support to New York City Health and Hospitals (H+H) in 2018 for implementation of peer and licensed clinician substance use ED intervention teams, known as the “ED Leads” teams. ED Leads teams provide substance use intervention services, including Screening Brief Intervention and Referral to Treatment and peer support for SUD treatment at 11 H+H EDs [35]. The purpose of this study is to (i) describe the ED Leads blended licensed-clinician delivered Screening Brief Intervention and Referral to Treatment and PC team model in the ED at three hospitals, (ii) provide a descriptive analysis of patient engagement and referrals to SUD care post-ED Leads intervention, and (iii) highlight potential barriers and facilitators to implementing the integrated model. This descriptive study adds to the dearth of literature on integrated Screening Brief Intervention and Referral to Treatment and peer support models in the ED for risky substance use and SUD.

## Methods

### Context

Three of the 11 hospitals funded for the ED Leads program were included in this study. Five hospitals were excluded because they had not yet hired or implemented the blended team, and so did not align with the combined service model in this study. Two facilities had not converted to the new electronic medical record (EMR) system during the study period and therefore data was unavailable for extraction. One facility predominantly provided psychiatric services and therefore was also outside of the scope of this study. The remaining three H+H ED Leads teams described are in the Brooklyn and Queens boroughs of NYC, referred to as Brooklyn Hospital 1, Queens Hospital 1, and Queens Hospital 2. Of note, Brooklyn Hospital 1 provided a small amount of licensed-clinician only services in the ED and other areas of the hospital prior to official launch of the program, but the authors determined that the impact was minimal.

While all facilities had an SUD outpatient treatment program, providing counseling and medication for addiction treatment (MAT), inclusive of medications for AUD, the location of the SUD outpatient treatment program and other on-site services varied. All sites had an outpatient treatment program which provided counseling and non-methadone MAT. Brooklyn Hospital 1 also had an on-site detoxification unit where patients could be admitted directly from the ED, and the SUD outpatient treatment program was off-site. Queens Hospital 1 also had an Opioid Treatment Program (OTP) which provide methadone and other SUD outpatient treatment program services. Queens Hospital 1 also launched a substance use consult service for the inpatient medical floors in July of 2019.

### Staffing

During the study period, which encompassed the first 120 days of implementation at each of the three hospitals, the team composition varied because of the length of time required for hiring and staff turnover (Table 1). All ED Leads teams provided coverage Monday through Friday from early morning into the evening. There was some variation in weekend coverage at most EDs, and one hospital provided some overnight coverage. ED Chiefs of Service and key ED stakeholders recommended shift days and times according to perceived times of greatest need (Table 2). ED Leads team members were supervised by members of their hospital’s SUD outpatient treatment program, but were dedicated to providing services in the ED. PCs were expected to be Certified Peer Recovery Advocates in alignment with New York State regulations, or to obtain certification shortly after hire.

**Table 1:**
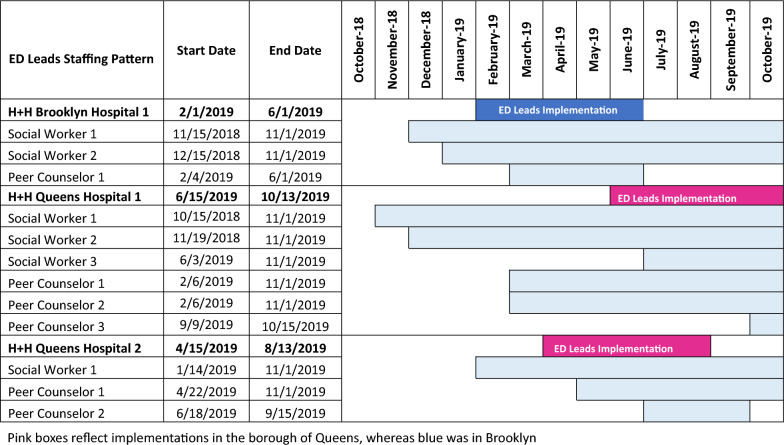
Hiring and implementation phase timelines

**Table 2:**
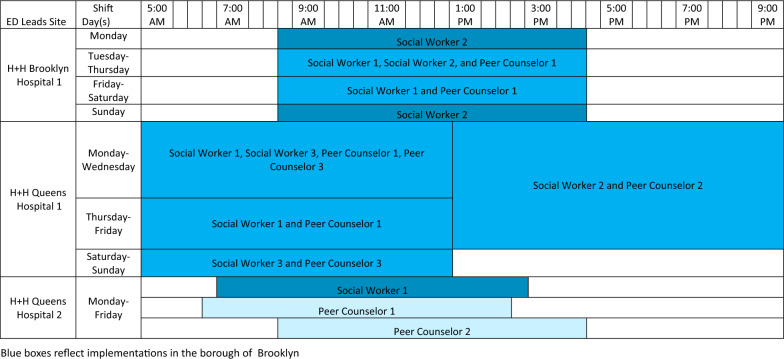
Staffing patterns

### Training

At orientation, ED Leads staff were provided with an ED Leads Scope of Program manual comprised of an implementation checklist, team schedule, and clinical and documentation workflows. Clinical workflows provided guidance based on scopes of practice and hospital operations, and were not highly protocolized. Staff participated in a naloxone and overdose education training, as well as compliance/patient-centered trainings on Health Insurance Portability and Accountability Act and Preventing and Managing Crisis Situations. Staff also participated in multiple EMR workflow trainings. On an ongoing basis, ED Leads were provided with technical assistance from an off-site H+H ED Leads project manager. Technical assistance included workflow optimization as well as facilitation of a re-occurring multi-disciplinary workgroup meeting with representation from the ED, Social Work, ED Leads, and SUD hospital leadership to maximize ED Leads service delivery.

### Patient engagement

ED Leads teams primarily used two lists in the EMR to case find. Potential ED patients would automatically populate onto a list if they met the following criteria: (1) screened positive on the Single Item Screening Questionnaire (SISQ) [36] for risky substance use (completed by ED nursing staff during triage); and (2) had a history of an SUD documented on their EMR problem list. Additionally, all teams carried either a pager or phone where ED providers could contact them for direct referrals.

Licensed clinicians followed the Screening Brief Intervention and Referral to Treatment model, including a screening with an evidenced-based tool such as the Alcohol Use Disorders Identification Test [37] or Drug Abuse Screening Test [38], followed by a brief intervention and referral to treatment, as appropriate. In most instances, an evidenced-based screening tool was not necessary because patients were previously screened, were identified as having risky substance use by ED staff, or the clinicians found that the patient had a history of risky substance use. PCs relied on the latter given evidenced-based screening tools are not in the scope of a peer interaction. Once identified, teams would attempt to engage in an intervention to educate the patient about their substance use and empower them to change their behavior. This may have included overdose prevention education and provision of a naloxone kit. Translation services were available to ED Leads remotely by phone or in person at bedside, at any time, and in more than 200 languages and dialects, including Spanish. Patients were offered a resource list of treatment services, and if feasible, were given assistance with making an appointment with a treatment program, a “warm referral”. ED Leads teams collaborated with the ED medical team to communicate arrangements for follow up substance use services, relay interest for MAT, and any other emergent needs outside of the ED Leads scope.

### Analysis

Patient EMR data was used, including a review of ED Leads team member progress notes documented during the first 6 months of services. Each patient visit to an ED was counted as unique, so patients may be counted multiple times if they had multiple ED visits throughout the study period. ED Leads staff’s EMR documentation was coded by the staff type writing the note (licensed clinician or PC). Next, documentation was coded for success in engaging a patient in PC or Screening Brief Intervention and Referral to Treatment services, as well as referrals provided to further SUD treatment. Successful engagement meant the patient was at a minimum willing to engage in an initial conversation with the ED Leads team member about substance use, irrespective of if the patient later accepted a referral to treatment or resources. Unsuccessful patient engagement included patients who refused to speak with the ED Leads team member or were unable to engage (i.e., were sleeping, incapacitated, or left before contact could be made.)

Those patients who were discharged from the ED and received a successful engagement were coded according to their referral type. Referrals were coded as “Warm Referral”, “Resource List” or “Not Referred”. We excluded patients admitted to the hospital’s inpatient medical service because they were no longer ED patients and were unable to accept a referral. Patients who were engaged by ED Leads team members in a conversation, but refused a “warm referral” or an SUD services resource list were coded as “Not Referred”. Patients who did not accept a “warm referral” but did accept a resource list, were coded as “Resource List”. Finally, patients who accepted a “warm referral” were coded according to whether that referral was to one of the seven H+H facilities utilizing the same EMR system (“Referred to H+H”), or one of the H+H facilities not using the same EMR system or facilities outside of the H+H system (“Referred to Community”). Of note, follow up for patients “Referred to Community” was unknown. Charts for patients “Referred to H+H” were reviewed for any follow up SUD care at H+H facilities within 7 days of engaging in an ED Leads encounter. These patients were further classified into two categories: “No SUD Treatment”, or “SUD Treatment”.

### Ethics

Data for the ED Leads program was collected as part of standard quality improvement (QI) processes at H+H. The H+H Research Office confirmed that the protocol for this study met QI criteria and did not need review by an institutional review board. Descriptive analyses and reports to leadership are regularly completed for QI purposes to guide service processes and modifications.

## Results

Across facilities, there were 1785 patients approached by ED Leads staff during the study period (Table 3). Patients were predominantly male (N = 1438, 81%) with an average age of 45 (SD = 13), enrolled in Medicaid (N = 1062, 59%), and had a diagnosis of AUD on their problem list (N = 801, 45%). Overall, 74% of patients (N = 1326) had a SUD documented on their problem list. SUD diagnoses for Queens Hospital 2 patients were largely missing in the EMR (N = 152, 42%), with AUD as the second largest group at that facility (N = 127, 35%). A majority of patients self-identified their race as “Other” (N = 761, 43%). A majority of patients at Queens Hospital 1 (N = 354, 55%) identified their ethnicity as Hispanic, in contrast to a minority at Brooklyn Hospital 1 (N = 162, 21%) and Queens Hospital 2 (N = 68, 19%).

**Table 3:** Patient demographics

	Brooklyn hospital 1		Queens hospital 1		Queens hospital 2		Total	
	N	%	N	%	N	%	N	%
Emergency department admissions	29,157		31,396		32,746		93,299	
Total patients approached	783		644		358		1785	
Sex								
Female	140	18	114	18	93	26	347	19
Male	643	82	530	82	265	74	1438	81
Age (M ± SD)	46 ± 12.6		43 ± 13.2		46 ± 12.9		45 ± 13	
Race								
American Indian/Native Hawaiian/Pacific Islander	6	1	2	0	1	0	9	1
Asian	18	2	45	7	18	5	81	5
Black	115	15	65	10	115	32	295	17
Other	189	24	402	62	170	47	761	43
Unknown	11	1	23	4	25	7	59	3
White	436	56	107	17	29	8	572	32
Ethnicity								
Hispanic	162	21	354	55	68	19	584	33
Non-Hispanic	545	70	233	36	242	68	1020	57
Unknown	76	10	57	9	48	13	181	10
Insurance								
Commercial	173	22	63	10	96	27	332	19
Medicaid	507	65	393	61	162	45	1062	59
Medicare	70	9	38	6	31	9	139	8
Other	4	1	8	1	6	2	18	1
Uninsured/self pay	0	0	142	22	0	0	142	8
Diagnoses								
AUD^a^	330	42	344	53	127	35	801	45
Other SUD^b^	0	0	3	0	0	0	3	0
OUD^c^	51	7	12	2	11	3	74	4
Poly SUD	300	38	80	12	68	19	448	25
Unknown	102	13	205	32	152	42	459	26

^a^Alcohol use disorder

^b^Substance use disorder

^c^Opioid use disorder

**Fig. 1:**
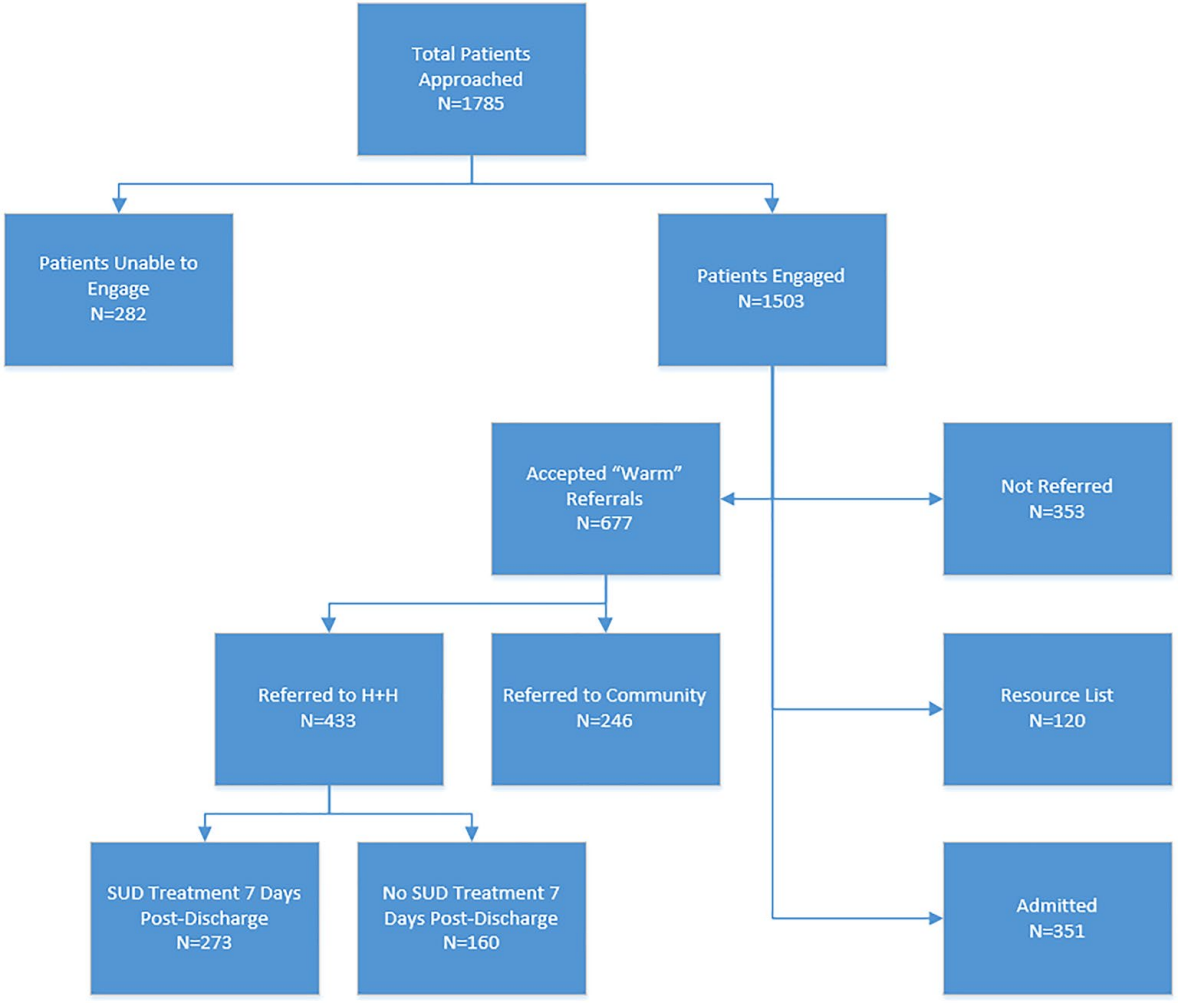
Patient encounter flow chart

Eighty-four percent (N = 1503) of patients approached by team members across sites were engaged (Table 4, Fig. 1). Of those, the preponderance were engaged by the clinician only (81%, N = 1221), with only 6% (N = 86) engaged by both ED Leads staff types. Of the patients engaged, there was wide variability between facilities regarding patient disposition at ED discharge. At Brooklyn Hospital 1 (N = 772), 42% (N = 321) were referred to one of the seven H+H facilities for SUD services, compared to only 16% (N = 73) at Queens Hospital 1 and 15% (N = 39) at Queens Hospital 2. At Queens Hospital 1, patients were most often not referred (N = 189, 40%), while at Queens Hospital 2 they were predominantly referred to the community (N = 113, 43%), and two were referred to non-SUD resources which were excluded from Table 4 and Fig. 1. Of note, 8% (N = 120) of all patients agreed to a resource list but not a referral, and 23% (N = 351) of patients were admitted to the medical or psychiatric hospital floors.

**Table 4:** Patient encounter details

	Brooklyn hospital 1		Queens hospital 1		Queens hospital 2		Total	
	N	%	N	%	N	%	N	%
Patients approached	783	44	644	36	358	20	1785	100
Patients unable to engage	11	1	176	27	95	27	282	16
Patients engaged	772	99	468	73	263	73	1503	84
Licensed clinician only	754	98	300	64	167	63	1221	81
Peer only	18	2	82	18	96	37	196	13
Both	0	0	86	18	0	0	86	6
Referrals								
Not referred	131	17	189	40	33	13	353	23
Resource list	64	8	18	4	38	14	120	8
Admitted	176	23	135	29	40	15	351	23
Accepted “warm” referrals	401	52	126	16	150	19	677	45
Referred to community	80	10	53	11	113	43	246	16
Referred to H+H	321	42	73	16	39	15	433	29
Treatment attendance 7 days post-discharge								
SUD^a^ treatment	256	80	16	22	1	3	273	63
No SUD treatment	65	20	57	78	38	97	160	37

^a^Substance use disorder

Of the patients referred to H+H SUD treatment programs (N = 433, 29%), 63% (N = 273) received follow up SUD treatment services within 7 days of the index ED admission. Note each admission was counted as a unique index ED admission and subsequently unique ED Leads encounter. Most notably, 58% (N = 252) received detoxification services, which were primarily patients seen at Brooklyn Hospital 1. Queens Hospital 1 had the largest number of outpatient follow-up visits (N = 16, 22%) while Queens Hospital 2 had the largest number of patients who did not receive SUD treatment services within 7 days at H+H (N = 38, 97%).

## Discussion

The ED Leads teams present a novel opportunity to rapidly engage patients and link them to substance use treatment, as appropriate, during an ED visit. The integration of dedicated staff specializing in addiction treatment, to relieve ED staff strain, filled a significant gap in SUD specific interventions in this service area. This study contributes to the limited literature on such programmatic innovation. Patients were generally willing to engage with ED Leads with less than 20% of patients referred to the teams not engaged during their ED presentation. Regardless of SUD treatment referral acceptance, patient engagement with ED Leads teams may support treatment initiation at a later date, sufficiently address risky substance use, provide overdose prevention education, or build rapport with team members for subsequent encounters during ED readmissions.

The majority of patients, however, did not meet with both a PC and licensed clinician, suggesting the blended model was not fully implemented as envisioned. Table 5 provides a summary of potential facilitators and barriers for implementation of a blended model. For example, Brooklyn Hospital 1 and Queens Hospital 2 did not have any patients who met with both staff types. There are potential implementation challenges that may underline this finding, as well as several nuances that may not be captured in the data. First, not all attempts to approach patients may have been documented, given the variability across sites of patients approached but not engaged, particularly between Brooklyn Hospital 1 and the other two sites. Additionally, staff may have documented only one note in the EMR per patient, even if both staff types met with the patient. Training should be clear that attempts and successful engagements need to be documented by each team member in a progress note.

**Table 5:** Potential facilitators and barriers for blended model implementation

Potential facilitators	Potential barriers
Institutional “buy in” to integrate PCs and licensed clinicians via financial support and stakeholder engagement	Need for specialized onsite supervision and oversight of staff to enhance model implementation
Clarity in program mission	Unclear workflows, roles and responsibilities among team members
Positive integration in ED^a^ due to the model filling the gap in ED substance specific interventions (i.e., dedicated staff to relieve ED staff strain)	Hiring and retaining PCs
Staff’s willingness to integrate and work collaboratively as a team	Navigating logistics of PCs^b^ and clinicians consistently engaging all patients due to ED time and workflow demands
	Variability and limited training for PCs in this specific ED role

^a^Emergency department

^b^Peer counselors

Staff roles, responsibilities, and workflows were also not fully protocolized during implementation. The mission of the program was likely clear to staff: engage as many patients as possible and facilitate referral to treatment. At implementation, best practices in the field were nonexistent and staff were not expressly told that every single patient needed to be offered services by both disciplines. Much of the blended approach relied on general guidance regarding known scopes of practice and hospital operations, but teams were left to their own creativity and experiences to see what worked and what did not for each patient. ED Leads staff may have divided their patient load to maximize time and outreach, which at the outset seems like a practical way to engage as many patients as possible. Additionally, this approach may have also been adopted given the challenging logistics and workflow demands of the ED environment. If a truly blended approach is defined as a patient being offered engagement from both types of staff, perhaps more clear protocols are needed to guide staff. This includes specifying that all patients should be offered engagement with both disciplines, and training staff on the benefits that each discipline brings to patient engagement.

The ability for ED Leads staff to fully incorporate their diverse skillsets may have been limited by staff retention and recruitment challenges. Queens Hospital 1 had the greatest number of PC positions filled for the longest duration and the most dual-staff encounters with patients. Queens Hospital 2 PCs had slightly more peer-only engagements comparatively despite having less PCs. This difference across sites could be due to differences in individual PC performance and training characteristics. There were also significantly more days during the implementation period with licensed clinicians hired compared to PCs, limiting the opportunity for the full complement of staff to be implemented. Supervisors of team members were also new to supervising teams in the ED and more generally PCs. Supervision structures were also not standardized across the sites, while some sites had supervisors as part of the team, other supervisors were in a different area of the hospital. Perhaps a dedicated and specialized supervisory structure may enhance blended model implementation.

While the ‘divide and conquer’ approach loses the nuance that both disciplines bring when engaging a patient, the staff’s willingness to work collaboratively may have been a potential facilitator for the model. Case consults may have been conducted to assist one another, but also to triage who might be a best fit based on known patient presentation, history, and any prior encounters. One could argue that this was a blended model that uses a more patient-centered approach, not discretely captured in the data. It is possible that not all patients would be interested in engaging with both staff types, and some may feel more comfortable interacting with one staff type vs. another. Future research could consider examining variations of a blended model that considers patient preferences for PCs or licensed clinicians, as well as barriers to, and facilitators of implementation including staff recruitment, retention, training, and team-building.

With regards to patient outcomes, there was wide variability across hospitals in the frequency and types of referrals provided to SUD treatment. The greatest number of 7-day follow ups was found to be detoxification, which may have different linkage rates, and ultimately, patient outcomes, than outpatient services, in part because patients can be directly admitted. The majority of patients at Queens Hospital 1 were either not referred, or were admitted to a more acute level of care. Since follow up for admitted patients is out of scope for ED Leads and the patient disposition at discharge is unknown, the assumption is that those are not ideal patients to target for a brief intervention. While it may not always be possible to know if a patient will be admitted at the time of ED Leads engagement, these findings raise the question regarding best strategies for case finding or mechanisms of referrals. For example, perhaps team members at that site needed to further refine their chart review process, and proactively communicate with the medical teams ahead of approaching patients to determine if they will be admitted. Additionally, medical teams may benefit from additional training to identify patients in need of engagement by ED Leads.

In comparison to the other sites, Queens Hospital 1 had the largest percentage of Hispanic patients, as well as the largest percentage of patients who were uninsured. Culturally and ethnically diverse patients often have limited or unsatisfactory options for community SUD treatment that can accommodate their language preferences, particularly considering the majority of treatment involves individual and/or group counseling. This may, in part, offer an explanation as to why so many patients at Queens Hospital 1 were not referred or did not accept a treatment referral compared to other sites. Separately, while H+H provides care regardless of insurance status, the large proportion of uninsured patients may have been reluctant to accept a referral due to fear of a potential financial burden. For the uninsured who may be undocumented, system level barriers to care have been identified such as long waiting lists, and some community programs’ unwillingness to admit uninsured patients, which contribute to the existing racial/ethnic disparities related to substance use treatment utilization [39]. This study found that a larger proportion of patients with commercial or subsidized insurance received a referral compared to those without insurance. Fear of potential stigma and discrimination from health care professionals can be a barrier to receiving traditional treatment [40]. As an effort to address such barriers, systems of care can employ PCs and substance use clinicians to broaden the provision of care in the ED, with potential to yield improved patient outcomes.

This study had several limitations. First, this study was descriptive and for quality improvement purposes, thus was not designed to establish effectiveness of the intervention. Additionally, results are based on staff documentation and therefore are prone to omissions and human error. It is not possible to identify case consults or the number of patients offered both PC and licensed clinician interactions during their ED visit. Further, EMR data only provides some elements of the encounter, and not the full scope of factors contributing to the outcome of that interaction. This is particularly true for the PC interactions, for which the impact of the encounter may not necessarily result in acceptance of a referral, which can be discretely measured, yet may still be of significant value to the patient for recovery. There are a number of patient and system level factors that influence one’s initiation, engagement, and adherence to treatment, such as patient demographics, immigration status and the amount of time between hospital discharge and the first treatment appointment, which were not examined in this study [41, 42]. Future research should consider metrics for success of an ED-based substance use intervention beyond the patient’s acceptance of a referral, such as patient-clinician rapport building during repeated ED presentations, patient’s report of a reduction in their substance use, patient’s engagement in opioid overdose prevention education, acceptance of an MAT consultation during the ED encounter, and ED readmission rate within a defined time frame. The study also did not take into account successful referrals to treatment establishments outside of the EMR system, or examine whether patients received MAT during the ED visit, or its connection to the ED Leads team intervention. Lastly, this study was unable to consider the time of day of the patient intervention, which could significantly impact connections to care since most appointments can only be made during business hours. Future studies should consider a thorough examination of the effectiveness of this model as well as the barriers and facilitators to implementation at multiple levels (i.e. patient, hospital, system and community).

## Conclusion

There is an ongoing need to utilize the ED for screening, treatment of SUD, and linkage to care [43]. Yet, addiction treatment expertise is an often under-resourced service in EDs. This descriptive analysis of the ED Leads program contributes to the scant literature available on non-traditional ED-based substance use interventions with a complex patient population, and aligns with the increased focus on recovery-oriented services in the field. This model is an integrated and promising approach at targeting this need, solidifying processes for screening and the provision of a direct referral to treatment during an ED visit. These findings highlight the importance of the hospital system’s use of organizational strategies and interdisciplinary integration to fill this gap in ED services. Though the engagement and linkage outcomes were promising, the successful implementation of a fully blended PC and clinician model was not supported as there were a number of unexpected barriers in its implementation. Barriers included limited and possibly incomplete data from staff documentation, unrefined workflows, and workforce challenges. The exact design of a peer integrated ED substance use intervention that is most effective at targeting an array of patient health and engagement outcomes remains unknown [12]. Further research is needed to fully understand the implementation and ultimate effectiveness of peer integrated models; however, this study may serve as an example for healthcare systems aiming to build and implement such novel services.

## Data Availability

The datasets generated and/or analyzed during the current study are not publicly available due to identifiable personal information that may not be released in compliance with applicable state and federal regulations.

## Abbreviations

NYC: New York City
SUD: Substance use disorder
OUD: Opioid use disorder
MOUD: Medications for opioid use disorder
ED: Emergency department
AUD: Alcohol use disorders
PCs: Peer counselors
H+H: New York City Health and Hospitals
EMR: Electronic medical record
MAT: Medication for addiction treatment
OTP: Opioid Treatment Program
SISQ: Single Item Screening Questionnaire
QI: Quality improvement
